# Behavioral change: from neurobiology to behavioral-change theories and back – part 1 *

**DOI:** 10.1055/s-0046-1825762

**Published:** 2026-08-31

**Authors:** Conrado Regis Borges, Fabiano Moulin de Moraes, Marcelo Youngblood, Felipe Chaves Duarte Barros, Nancy Huang, Marcos Christiano Lange

**Affiliations:** 1 Faculdade Sírio-Libanês, São Paulo SP, Brazil.; 2 Universidade Federal de São Paulo, Departamento de Neurologia, São Paulo SP, Brazil.; 3 Universidade Estadual de Ponta Grossa, Ponta Grossa PR, Brazil.; 4 Universidade de São Paulo, Departamento de Neurologia, São Paulo SP, Brazil.; 5 Universidade Federal do Paraná, Departamento de Neurologia, Curitiba PR, Brazil.

**Keywords:** Health Behavior, Behavior Therapy, Motivation, Health Behavior, Preventive Medicine, Health Education, Neurobiology

## Abstract

**Background:**

Behavioral change remains challenging despite a vast body of evidence linking health behaviors with several health outcomes. Over the past few decades, various health-behavior change theories and techniques have emerged, leading to significant advances in the field. However, although some existing theories provide competent models to structure behavioral-change approaches, they do not provide a unified neurobiological foundation.

**Objective:**

To outline the connections between neuroscientific foundations and behavioral-change theories and to provide an overarching neuroscientific perspective.

**Methods:**

We conducted a structured narrative review based on data retrieved from major biomedical databases and authoritative neuroscience textbooks to integrate neuroscience data with behavioral-change theories.

**Results:**

We describe the neuroscience of behavior from neuroanatomical to neurophysiological mechanisms, emphasizing four brain systems: the hypothalamus, the amygdala, the mesocorticolimbic system, and the prefrontal cortex. Finally, we integrate these mechanisms with a neuroscience-based motivational theory called the Behavior Change Resource Model.

**Conclusion:**

The present work integrates established neurobiological knowledge and behavioral-change theories, providing a framework to link theory and health-behavior interventions.

## INTRODUCTION

Lifestyle and behavioral factors influence the risk of developing chronic diseases and their complications. 1 Concepts, such as the Life's Essential 8 2 to promote cardiovascular health and, more recently, Brain Health, 3 have emerged to emphasize the importance of lifestyle factors in maintaining health.

Lifestyle is a combination of individual behaviors that can be either adverse, neutral, or beneficial to health. 4 Although the benefits of stopping damaging behaviors and developing beneficial ones have been well established, 5 successfully modifying behaviors and improving lifestyles remain challenging for patients and healthcare professionals.

Health behaviors are complex phenomena. 6 Making changes to physical activity, diet, and sleep habits requires multiple and, at times, competing reasons. 7 It typically demands self-regulatory skills and a shift in how individuals guide themselves in their environment. 8 Smokers, for example, are frequently aware of the risks associated with smoking cigarettes. In any given year, 30 to 70% of current smokers attempt to quit smoking, but few succeed. 9 Similarly, many people set goals to pursue other health-behavior changes, such as eating healthier and losing weight; however, most fail to achieve their resolutions despite being confident they will succeed. 10

The high failure rate in achieving desired changes demonstrates how complex it is to initiate and maintain behavioral change. 6 11 Beyond their complexity, behavior is frequently influenced by other factors. 12 13 For instance, many people drink alcohol to cope with negative feelings or to relax in stressful occasions. Increasing physical activity involves feeling discomfort, such as sweating and muscle pain, and spending less time on other activities, like resting after a stressful day in front of the TV. 7 14

Changing health behaviors may also involve changes in social dynamics (for example, replacing an after-dinner smoke with going out with one's partner to walk) and in norms and traditions (such as adapting food recipes to meet new goals), with possible pushback from one's community. 15 16 Making the change, thus, is both social and personal. 17

Another facet is that problematic behaviors frequently co-occur. 18 Individuals who smoke, for example, are more likely to be less physically-active and to have a poor diet. Besides this connection, individuals typically overlook how one behavioral change affects other health behaviors. 19 A typical example is quitting smoking, which may lead to weight gain, which, in turn, may hinder smoking cessation attempts. Furthermore, many individuals focus primarily on initiating health-behavior change without diligently considering the importance of maintaining those behaviors over time. 20 21

In summary, behavioral change is often difficult in real-world settings. One effective way to address this complexity is to deepen our understanding of behavior and examine the main schools of behavioral science. In the present paper, we describe human behavior and its neurobiology, following several key approaches to behavioral change. In addition, we outline the connections between neuroscientific foundations and behavioral-change theories and propose an overarching neuroscientific perspective that bridges motivational theories and clinical behavioral-change strategies.

## METHODS

We chose the structured narrative review design for the current work because this approach enables the integration of a broad range of scattered neuroscientific literature to perform an interpretive synthesis and to support the development of a framework to organize neurobiological processes underlying behavioral change and psychological theories.

Initially, we mapped pertinent authoritative book chapters on highly-consolidated subjects, such as neuroanatomy and neurophysiology, in their most recent editions. Within these sources, we identified four neural systems that are consistently highlighted in the literature as relevant to behavioral change, which served as the conceptual axis of the present study: the hypothalamus, the amygdala, the mesocorticolimbic system (MCLS), and the prefrontal cortex (PFC). Additionally, we catalogued the foundational articles on each research domain.

Finally, we used the PubMed, Scopus, Web of Science, and SciELO databases to identify recent, relevant articles. We conducted searches from December 2024 to August 2025. The inclusion criteria were as follows: studies published in English; peer-reviewed scientific articles; original studies, meta-analysis, or reviews; and articles on a topic consistent with the objectives of the current review.

The databases were queried for the following terms across article titles, abstracts, and keywords, combined with Boolean operators. Two researchers (CRB and FMM) independently conducted the database searches using the predefined key terms. First, we searched for basic neuroscientific data using the following terms: *Neurobiology* OR *Neurophysiology* OR *Hypothalamus* OR *Frontal Lobe* OR *Amygdala* OR *Mesocorticolimbic System* OR *Prefrontal Cortex* . Then, we searched for applied-science topics: *Behavior* OR *Behavior Change* OR *Health Behavior* OR *Behavior Change Theory* OR *The Behavior Change Resource Model* OR ( *Motivation* AND *Neuroscience* ), OR *Health and Lifestyle Coaching* . Furthermore, we examined the citations and reference lists of the retrieved reviews to identify additional relevant publications.

## WHAT IS BEHAVIOR?

The literature does not provide a single, universally-accepted definition of behavior. However, in the current work, we will use thedefinition by the American Psychological Association: ‘the activities of an organism in response to external or internal stimuli, including objectively observable activities, introspectively observable activities, and unconscious processes.’ 22 From this perspective, behavior is understood as any response of an individual, regardless of the origin or expression of the stimulus. This broad approach facilitates a nuanced description of behavior.

**Figure 1:**
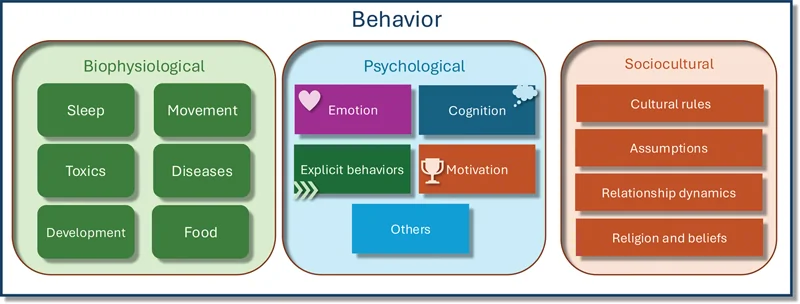
Levels of behavior and their corresponding domains: Biophysiological, psychological, and sociocultural. 13

Behavior can be interpreted across sociocultural, biological/physiological, and psychological levels ( Figure 1 ). 23 The sociocultural level involves a given culture's rules, assumptions, and conventions. The biological level addresses the changes the organism undergoes during an event across multiple systems (such as in neurotransmitters, hormones, heart rate, blood pressure, intestinal transit, and musculoskeletal changes). Lastly, the psychological level encompasses intrapsychic manifestations, such as sensations, perceptions, emotions, and thoughts experienced at any moment.

An illustrative example of behavior is the widespread difficulty in adhering to physical exercise in contemporary society. At the sociocultural level, professions that confine individuals indoors predominate, along with strong incentives for sedentary activities such as watching television or engaging with social media. At the biological level, physical inactivity correlates with poor physical fitness, weight gain, metabolic syndrome, and increased morbidity and mortality. At the psychological level, there is often a lack of motivation to initiate physical activity, linked to feelings of hopelessness and low self-efficacy.

This division of behavior into levels serves a didactic purpose; however, it is essential to note that these levels are deeply interconnected and mutually influential. The biological consequences of physical inactivity, such as weight gain, for example, can significantly impact the psychological level, leading to low self-esteem and feelings of hopelessness, as well as the sociocultural level, by fostering social exclusion. Additionally, reciprocal influences can also be observed.

Moreover, behavior formation is dynamic and continually evolves at each level throughout an individual's life. Personal experiences gradually shape behavioral patterns across various contexts. Over time, individuals develop beliefs, values, and habits that can be challenging to modify, depending on their psychological flexibility.

Effective models to assist behavioral change should consider all levels of behavior. The Behavior Change Resource Model, described in the following section, does so considering the wide range of determinants of behavior. Although it focuses on volitional and goal-directed behaviors, it also integrates them with non-volitional dimensions, such as affective drives and environmental factors.

## THE NEUROBIOLOGICAL FOUNDATIONS OF BEHAVIOR

Comprehending the (neuro)biological processes of behavior is fundamental, as all behaviors ultimately arise from the functioning of the central nervous system. The present work conceptually bridges fundamental neurobiological components of behavior with a model proposed by Michaelsen and Esch 24 that organizes most of the evidence-based processes that facilitate behavioral change.

**Figure 2:**
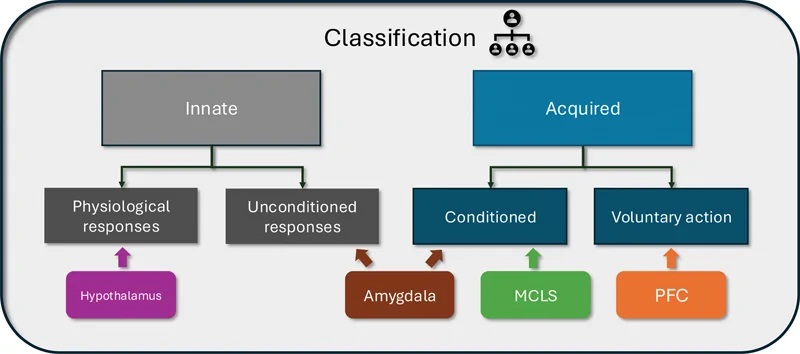
Brain structures involved in behavioral change.

Four brain structures are key to behavior generation ( Figures 2 3 ). The hypothalamus regulates primitive functions related to basic needs; the amygdala plays a central role in the management of aversive and appetitive responses (such as avoiding or approaching stimuli); the MCLS biases behaviors toward desirable outcomes (such as moving closer to stimuli); and the PFC supports more complex behavior management, such as structuring plans, delaying rewards, or inhibiting impulses. 25

**Figure 3:**
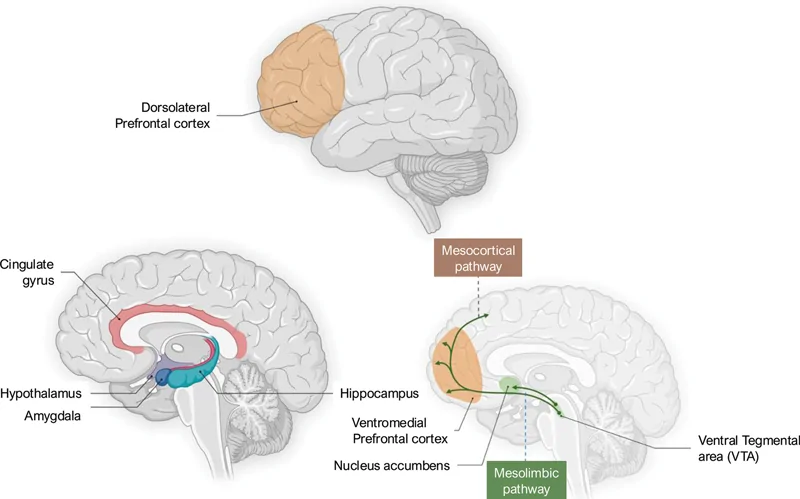
Abbreviations: MCLS, mesocorticolimbic system; PFC, prefrontal cortex. Classification of behavior based on acquisition type and corresponding structures.

### The hypothalamus

The hypothalamus ( Figures 2 3 ) serves as the central hub to regulate an organism's automatic responses and allostasis. Through its connections with the brainstem, it regulates the sympathetic nervous system (SNS) and the parasympathetic nervous system (PNS). In this context, it triggers ‘fight or flight’ responses by activating the SNS, increasing blood pressure and heart rate, causing sweating in the extremities, and leading to splanchnic vasoconstriction. Conversely, it initiates ‘rest and digest’ responses by activating the PNS, reducing blood pressure and heart rate, promoting splanchnic vasodilation, and increasing intestinal transit. 25

Additionally, the hypothalamus regulates the body's hormonal environment by stimulating the pituitary gland. For instance, it promotes the release of adrenocorticotropic hormone (ACTH), which ultimately leads to cortisol release in peripheral tissues. In conjunction with SNS activation, cortisol release is part of the stress response. 25 The hypothalamus also participates in thermoregulation and in the regulation of the sleep-wake cycle via the suprachiasmatic nucleus, synchronizing the biological clock with light-dark cycles. It further modulates the organism's appetitive responses, such as hunger, thirst, and sexual desire, increasing or decreasing the likelihood of an individual engaging in these behaviors. 25

External stimuli modulate its actions. Prolonged fasting, for example, is associated with increased ghrelin release, which can induce hunger sensations and modulate goal-directed behaviors to prompt food seeking. 26 Similar hypothalamic activation occurs in scenarios such as fluid deprivation (prompting liquid-seeking behavior), 27 sleep deprivation (increasing the drive to sleep), 28 and temperature changes (prompting clothing adjustments). 29 Situations involving difficult-to-change behaviors, such as caloric-deficit needs, tend to activate this system, 30 leading to challenges in behavioral control.

The hypothalamus receives input from alertness-associated nuclei, such as the locus coeruleus and raphe nuclei; the nucleus of the solitary tract, which conveys visceral information; the limbic system, particularly the stria terminalis and the ventral amygdalofugal pathway, providing emotional influences; and the PFC via the medial forebrain bundle, influencing hormonal release through the anterior pituitary. In turn, it sends outputs to the brainstem, exerting direct control over the SNS and PNS, and to the amygdala, hippocampus, insula, and PFC. 25

### The amygdala

The amygdala ( Figures 2 3 ) is a bilateral structure located in the medial temporal regions anterior to the hippocampus. 25 It is part of the limbic system and is traditionally associated with emotional responses to stimuli, 31 both aversive 32 (such as fear, anxiety, and anger) and positively-valenced. 33 In this regard, the amygdala assigns emotional valence to experiences, whether they are linked to avoidance or approach behaviors.

The connections of the amygdala with other structures illustrate its functions. It predominantly receives sensory input through two pathways. 25 34 The first is subcortical, also known as the *fast pathway* , which projects to the amygdala via the thalamus and midbrain. The second is cortical, referred to as the *slow pathway* , which projects to the amygdala through the temporal lobe, carrying information derived from various perceptual modalities.

The fast pathway is associated with the startle response, in which an unexpected event rapidly triggers an emotional reaction before comprehension occurs. 34 The slow pathway is linked to emotional responses that occur after more complex cortical networks process the information. 34 Numerous inputs reach the amygdala via this pathway, including semantic information about objects, pain signals, and aversive perceptions from the insula (such as those related to spoiled food). 35

Conversely, the amygdala sends efferent signals to several structures: the hypothalamus, which influences SNS activation and the hypothalamopituitary-adrenal axis, thereby generating autonomic and stress-related ‘fight-flight-freeze’ responses; 24 the hippocampus, which impacts the formation of memories tied to emotional contexts; 36 and the nucleus accumbens, which contributes to assigning positive emotional valence to events. 25 36 Additionally, the amygdala has bidirectional connections with the PFC, 37 influencing attentional processes and goal-directed behaviors while modulating the orbitofrontal and ventromedial regions of the PFC.

The emotional responses managed by the amygdala can be classified as innate or acquired. The central nucleus, which contains the main efferent pathways to the hypothalamus and midbrain nuclei, mediates innate responses. Three primary classes of threats trigger these responses: aversive sensory stimuli, such as pain, shock, or suffocation; the presence of natural predators; and unfavorable interactions with individuals of the same species. Aversive social triggers are complex in humans and are perceived through verbal and non-verbal communication. 38 39

Acquired emotional responses arise through conditioning. These responses, which are essential for survival, enable individuals to build a personalized behavioral repertoire against threats. 31 The basolateral nucleus of the amygdala, which receives sensory inputs about the environment and connects bidirectionally with the PFC, hippocampus, and mesolimbic system, mediates the acquisition of emotional responses, essentially assigning emotional value to experiences as either negative or positive. 25

The amygdala's role in fear conditioning is demonstrated in studies 32 using animal models of Pavlovian classic conditioning. In the classic experiment, researchers paired an electric shock (an unconditioned stimulus) with a stimulus that typically does not elicit an emotional response, such as a sound (neutral stimulus). After the initial association period, exposing the animal to the sound (conditioned stimulus) alone produces an aversive fear response (conditioned response).

This concept can be applied to behavioral change. Aversion to physical activity is a common example. In a hypothetical clinical scenario, a sedentary patient with fibromyalgia is encouraged to begin exercising. Inadvertently, the patient starts exercising at a high frequency and intensity. Days later, the patient experiences a significant worsening of pain, which triggers anxiety and persistent thoughts. These factors, combined with the pain, lead the patient to discontinue the behavioral-change attempt. In future situations, when the patient is once again encouraged to engage in physical activity, they may experience the same anxiety felt previously, even without the recurrence of pain, reducing the likelihood of engaging in the behavior. This phenomenon can also occur in other scenarios relevant to lifestyle medicine, such as attempting to quit smoking and dealing with insomnia ( Table 1 ).

**Table 1:** Common scenarios in lifestyle medicine and their connections to aversive conditioning

Behavior	Aversive trigger
Physical activity	Pain, fatigue, effort
Diet change	Loss of individual and cultural identity, social isolation
Weight loss	Loss of a negative reinforcer * , social isolation
Quitting smoking	Social isolation, loss of a negative reinforcer *
Quitting drinking	Social Isolation, loss of a negative reinforcer *
Sleep	Loss of a negative reinforcer * (e.g.: screens, late-night exercise)

Notes: *Negative reinforcement is the encouragement of certain behaviors by removing or avoiding a negative outcome or stimulus (eg: working to reduce anxiety, distress, sadness due to a frustrated marriage). It is different from positive reinforcement, which encourages behavior by adding a reward (working for personal or social gains).

### The dopaminergic system (MCLS)

The dopaminergic system ( Figures 2 3 ) consists of dopaminergic neurons located in the ventral tegmental area (VTA) of the midbrain, which connect to the nucleus accumbens (NAc)—the mesolimbic system—and structures of the PFC—the mesocortical system. 40 This broader network is known as the *MCLS* .

Experimental studies 40 have demonstrated that dopamine release in the NAc occurs following unconditioned events, such as food or water, as well as following conditioned stimuli. These findings have contributed to popularizing the association of the dopaminergic system with reward and pleasure. The NAc—especially the shell portion—sends efferent signals to subcortical limbic system structures, such as the hypothalamus and central amygdala, and it has been linked to assigning emotional valence to experiences.

However, a growing body of evidence 41 has challenged the link between dopamine and pleasure due to various inconsistencies. First, the concept of reward is broad and ambiguous. Additionally, studies using animal models that block the dopaminergic system have not reported a reduction in behaviors aimed at seeking reinforcing stimuli, such as food.

Conversely, the literature 24 associates the dopaminergic system with motivational processes involving approach. Although an individual's VTA releases dopamine in the NAc following a reinforcing stimulus, this response is significantly more intense when the positive outcome is unexpected (that is, in prediction-error scenarios). In other words, the dopaminergic system signals that a specific action has produced an unexpected desirable result and increases the likelihood of repeating the same behavior (reinforcement). 41

However, after the individual learns the association between the behavior and the positive consequence, dopamine release following the event decreases or even becomes absent. 42 43 Conversely, moments before the reward is received, significant increase in dopamine spikes occurs in association with the conditioned stimulus. 24 42 44 This phenomenon is known as *reward anticipation* . An illustrative real-world example is the anticipation of a highly-awaited movie premiere. In the days and weeks leading up to the premiere, fans engage more on online forums, search Google for additional information, pay higher prices for advance-screening tickets, and purchase more merchandise. Furthermore, the box office results from the opening weekend strongly predict the film's overall sales performance. 45

The predictability of a rewarding event can modulate reward anticipation. Intermittent rewards, especially when delivered randomly, have been associated in experimental settings with higher behavior frequency and increased dopamine release. This phenomenon is known as *random intermittent reinforcement* . Experimental studies 46 involving animals and humans have shown that the likelihood of target behaviors was much higher when the reinforcement was random rather than predictable. This principle can be observed in casinos 47 and on social media platforms. 48 Both scenarios use this type of reinforcement to increase target behavior. Casinos employ monetary rewards to increase the frequency of betting behavior. Social media platforms provide personalized, engaging content to extend the time spent on the app, consequently boosting ad exposure.

The MCLS mechanisms contribute substantially to promoting and refining goal-directed behaviors. This system enables the gradual development of complex motor skills, such as walking, speaking, writing, and playing sports, as well as cognitive skills, such as performing calculations and structuring reasoning. The dopaminergic system biases behavior toward desired goals, enabling it to be gradually refined and automated. 49 Conversely, habituation occurs as the behavior consolidates, thereby reducing its involvement.

A remarkable issue is that, despite its essential role, the MCLS can act as a barrier to adopting healthy lifestyle habits. In contemporary society, easily-accessible or artificially-enhanced reinforcers (such as ultra-processed foods, social media, and entertainment) are overwhelmingly available. Through a mechanism similar to addiction, they hijack the MCLS and hinder deliberate decision-making. 50 51 Furthermore, these behaviors, once quickly learned, become automated. However, this system can be harnessed if appropriately directed to foster healthy behaviors. To this end, practitioners can apply strategies from behavioral sciences described in the following sections.

### The prefrontal cortex

The three systems discussed so far share a common feature: they produce fundamentally-automatic responses that demand relatively-low energy. Additionally, in the cases of the amygdala and the MCLS, this process of automation arises from conditioning based on the individual's experiences. The function of the fourth system differs, as it is associated with voluntary responses. The brain structure responsible for these functions is the PFC ( Figures 2 3 ).

The PFC is a large structure with higher energy demands compared to most other brain regions. It is considerably more extensive and complex in humans than in other mammalian species, including non-human primates. Its functions have had significant adaptive value, enabling tool production, initiating and maintaining complex social interactions, and fostering self-awareness. 52 The PFC is often described as the brain's executive center. It plays a central role in adaptive behavior, enabling individuals to interact flexibly with their environment. It also enables the retention and manipulation of representations of the past, present, and future. 52 53 It can be divided into two broad regions based on functionality: the dorsolateral/dorsomedial PFC (dl/dmPFC) and the orbitofrontal/ventromedial PFC (of/vmPFC). 52

The dl/dmPFC is directly involved in cognitive control. It initiates and manages behaviors in context to achieve specific goals, known as *goal-directed behaviors* (GDBs). It plays a role in attention management and working memory. Additionally, it executes higher-order cognitive functions, such as logical thinking and abstraction. 52 The of/vmPFC is associated with behavioral regulation. It influences motivation, emotional regulation, vegetative states, and social cognition. It enables individuals to behave appropriately when impulses point in the opposite direction, such as during times of stress or social dilemmas. 34 52

These GDBs are complex and rely on the activity of the two aforementioned PFC regions, supported by other neural networks (such as the temporoparietal associative area and the limbic system). They can be didactically unfolded in six stages: goal definition and maintenance; sequencing and planning; initiation; action monitoring and error detection; rule and criterion switching; and response inhibition. 34 These processes occur throughout wakefulness and operate continuously at various short- and long-term levels.

In a hypothetical and illustrative scenario, preparing a whole-grain sandwich with ricotta is a GDB in the short term. The individual establishes the goal (goal definition) of making the sandwich and initiates the process (initiation). From there, they sequence (planning) actions, such as opening the refrigerator to gather ingredients, getting a knife from the drawer, and taking bread from the pantry. During execution, they check whether the actions align with the plan and may notice that some planned steps are not as expected (error detection), such as finding no clean knife, which requires adding a new step to the process (plan switching). Finally, they must inhibit competing behaviors during the process (inhibition), such as the urge to substitute ricotta with jam or whole-grain bread with cake.

In the long term, GDBs can extend over significant periods, such as during behavioral changes. The challenge lies in maintaining the plan in spite of the emergence of various factors, such as stress, sleep deprivation, and decreased motivation. An effective strategy to enhance adherence to long-term goals is to break them down into smaller, achievable milestones that can be met regularly and consistently. 54 The benefits of achieving small goals appear to be linked to the PFC's interactions with other networks: achieving goals activates the MCLS, reinforcing the corresponding behavior. Conversely, the sensation of failure (or low self-efficacy) that arises when individuals perceive they have failed generates aversive physiological responses, processed by the amygdala, which reduces the likelihood of continuing the behavior.

### Connections between the PFC and other networks

The PFC exhibits peak activity in novel situations, 55 when there is a need to decide between conflicting behavioral options or to resist an impulse. 34 56 In such cases, the PFC must make a series of small decisions that involve a high cognitive load. However, the PFC is not limited to deliberate behavior; it also participates in behavior automation. Over time, as the PFC generates decisions and behaviors in similar situations, and the most adaptive responses tend to be selected and repeated, gradually becoming automated. 49 These automated responses are called *schemas* . 57 Automation significantly reduces the PFC's metabolic demand and cognitive load, enabling it to focus on general-level decisions.

Learning to drive is a classic didactic example of behavioral automation. Initially, driving demands significant attention to properly sequence the necessary actions. Over time, these actions progressively integrate until attention is mostly directed to uncontrollable variables, such as road signs, other vehicles, and pedestrians. But automation also occurs in daily activities, decisions, and thoughts, which can favor or hinder the establishment of healthy lifestyle habits.

The interaction between the PFC and MCLS plays a key role in this process ( Table 2 ). On the one hand, GDBs that yield desirable results produce dopamine spikes in the NAc, fostering the repetition of specific behaviors, decisions, or thoughts and biasing individuals toward particular goals. 49 On the other hand, behaviors that generate undesirable (aversive) responses are associated with dopamine release in the vmPFC, activating error-correction mechanisms. 58 In this context, the dopaminergic system can be conceptualized like a dual filter within the PFC, biasing GDBs toward the most relevant goals and helping to inhibit behaviors that lead to undesirable outcomes. In addition, the of/vmPFC region has dense connections with the amygdala and modulates its responses across various functions, such as assigning positive values, processing aversive responses, and social communication. 37

**Table 2:** Neurobiology of behavior: integrating everything

**Figure 4:**
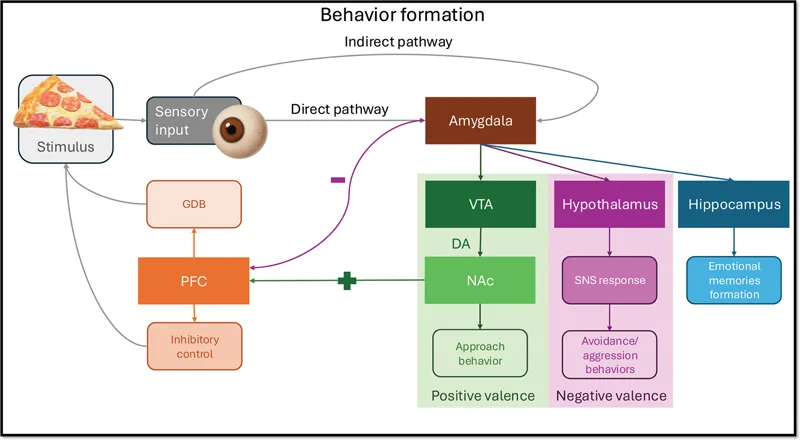
Abbreviations: DA, dopamine; GDB, goal-directed behavior; NAC, nucleus acumbens; PFC, prefrontal cortex; SNS, sympathetic nervous system; VTA, ventral tegmental area. Structures involved in behavior formation.

An individual's behavioral patterns are shaped by their interactions with the environment, which involve the structures and circuits mentioned in Figure 4 . The hypothalamus regulates involuntary responses of the autonomic nervous system, hormone release, and vegetative functions. It uses its connections with other brain structures to either increase or decrease an individual's predisposition to a specific behavior, ensuring the maintenance of homeostasis. 25 The amygdala assigns emotional valence to positive and negative experiences through its connections with midbrain structures. It labels events, objects, and experiences as *good* or *bad* , triggering cascades that may result in approach or avoidance behaviors toward the stimulus. 32 33 When a stimulus has a positive valence, the amygdala activates the ventral tegmental area (VTA), 25 the dopaminergic center of the mesocorticolimbic system (MCLS), which is associated with motivation. 41 When triggered by a reinforcing stimulus, the dopaminergic system increases the likelihood of repeating the same behavior, sometimes at the expense of others. Consequently, the most reinforced behavioral patterns tend to be repeated more frequently and become predominant. 49 The prefrontal crtex (PFC) is the brain's executive center for voluntary behaviors. It incurs significant energy costs when dealing with highly-complex demands. 52 The PFC generates goal-directed behaviors (GDBs) and inhibits maladaptive responses. It is highly-active in novel situations, such as behavioral change, but it reduces activity as contingencies become more predictable. 55 Its interaction with other brain systems, including the amygdala and the MCLS, is essential in alleviating cognitive load. Through its connections with the orbitofrontal/ventromedial PFC (of/vmPFC), the amygdala inhibits behaviors that have previously led to negative consequences. Conversely, the PFC can reduce aversive responses triggered by the amygdala. 52 The MCLS influences behavior by reinforcing specific actions, enabling their repetition and refinement. This process ultimately automates behavioral sequences and reduces the cognitive load on the PFC.

As aforementioned, the amygdala can assign positive values to objects. However, adaptive responses generally depend on the amygdala to dynamically update object values according to context. A chocolate bar, for example, holds much higher value when an individual is fasting than after eating five rows of chocolate. Updating the reward value is associated with bidirectional interactions between the amygdala and the ofPFC. 37 The ofPFC also modulates the amygdala's aversive response. Animal model studies 59 have shown that lesions destroying most of the ofPFC of monkeys increased avoidance responses and reduced approach behaviors toward threatening stimuli. This suggests that the ofPFC can produce negative feedback through its connections with the amygdala, attenuating aversive emotional responses. In this sense, the ofPFC plays a vital role in dampening defensive responses when the subject does not find adverse outcomes. This is in part attributable to the structure's capacity to process information about aversive stimuli. 37

In a hypothetical scenario, perceiving a shadow from behind while walking on a deserted street may initially trigger a reflexive aversive response, such as a startle. Within seconds, PFC involvement may facilitate attention to the stimulus source, revealing it as the shadow of a tree, thereby eliciting relief.

## THE PATHWAY TO BEHAVIORAL CHANGE

Several theories emphasize that behavioral change is a continuous, multistage process. Michaelsen and Esc 11 have recently proposed a model that combines features of widely used theories.

According to this model, the behavioral-change process consists of seven stages: unawareness, awareness, contemplation, planning, initiation, continued action, and maintenance. It resembles the widely-adopted Prochaska et al.'s transtheoretical model, 60 but it splits the precontemplation stage into two parts. In addition, it clusters the seven stages of change into three motivational engagement stages: non-engagement (unawareness and awareness), motivational engagement (contemplation and planning), and executive engagement (initiation, continued action, and maintenance). 11

In the non-engagement stage, the individual is neither motivated nor pursuing behavioral change. They remain comfortable with their current unhealthy behaviors despite the potential negative consequences. To describe this phenomenon, the authors 11 propose the theoretical construct of *assertion motivation* , which is linked to the non-wanting system and is characterized by inaction, acceptance, and a sensation of homeostasis ( Table 3 ).

**Table 3:** Assertion motivation

Most research does not contrast behavior driven by approach motivation and behavior driven by assertion motivation (AM), which is a theoretical construct that describes a “non-wanting” system, characterized by inaction, acceptance, contentment, and homeostasis. It entails the motivation to sustain a particular state with no propensity to move away from it. It differs from approach and avoidance motivation in terms of affective qualities, behavioral outcomes, and the non-activation of the dopaminergic system. Assertion motivation is associated with increased activity in the parasympathetic nervous system (PNS) and with neurotransmitters such as oxytocin, serotonin, endogenous opiates, endocannabinoids, and acetylcholine. Brain areas involved in AM, thus, include the vagus nuclei, midbrain, hypothalamus, pituitary gland, hippocampus, cingulum, and ventral striatum. 11 24 61

In the motivational engagement stage, the individual feels motivated to change but has not yet begun acting. The systems of approach motivation, processed by the MCLS, and avoidance motivation, processed by the amygdala, play key roles in guiding the individual toward healthy behaviors and away from unhealthy ones. Additionally, the individual's PFC systems begin to engage in the initial stages of the GDBs, such as goal-setting and planning, which will be relevant in the next stage. 11

In the executive engagement stage, the PFC's system for voluntary control, responsible for the GDBs, tends to increase its activity to enable an individual to remain engaged in health-behavior change. After initiation, some challenges may emerge, such as monitoring the process to avoid impulses toward unhealthy behaviors and adjusting strategies as needed. Since this stage demands high energy, the individual will typically require various resources, including the approach motivation drive. 11

If individuals succeed in maintaining healthy behaviors over extended periods, the interaction between the MCLS and PFC tends to gradually reinforce these behaviors, establishing new positive habits (automatic health behaviors). This reduces the PFC load without leading to disengagement. At this point, the individual returns to assertion-motivation mode, integrating the new behavior into their life.

## THE BEHAVIOR CHANGE RESOURCE MODEL

As previously stated, considering the neurobiological process of change is fundamental to design effective interventions for behavioral change.

**Figure 5:**
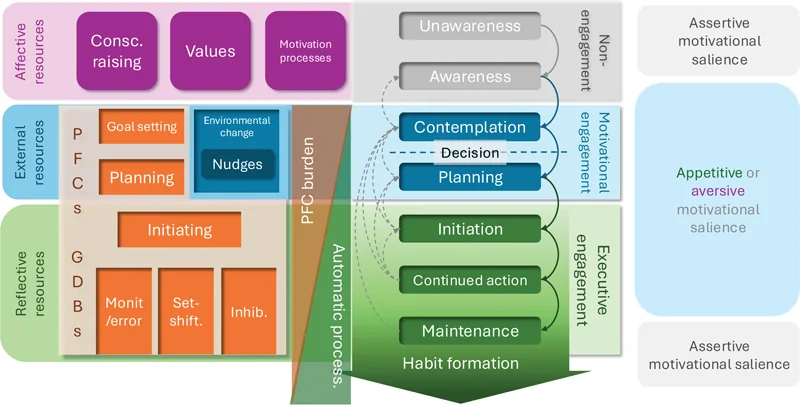
Abbreviations: Consc, consciousness; GDB: goal-directed behavior; Inhib, inhibition; Monit, monitoring; PFC, prefrontal cortex. Behavior Change Resource Model and its relation to the Model of Engagement. 41 .

The Behavior Change Resource Model, proposed by Michaelsen and Esch, 24 61 is a straightforward framework that organizes behavioral-change resources into three categories: internal affective resources, external resources, and internal reflective resources. 61 Each resource type is better suited, although not exclusively, to address one motivation engagement stage. 24 61 ( Figure 5 and Table 4 ).

**Table 4:** Three types of behavioral-change resources 24

Type of resources	Definition	Mechanism	Examples
Internal affective	Biological or psychological conditions referring to the individual's affective state	Activated by stimuli without intentional effort	Emotions (affects) and their reinforcements
External	Socioenvironmental conditions outside of the individual	Can be changed through intentional effort	Environmental and cultural context and material resources
Internal reflexive	Biological or psychological conditions referring to the individual's cognitive state	Generated, accessed or improved through effort and conscious deliberation	Goals and behavior regulation

In the current review, we extend the Behavior Change Resource Model by providing a neurobiological mapping of each resource and integrating the underlying systems. This mapping is conceptual and aims to facilitate integration between neuroscience and behavioral sciences, rather than to propose a strict one-to-one anatomical correspondence.

### Using affective resources to facilitate behavioral change

Individuals in the non-engagement stage are typically challenging to approach because they either do not realize that change is needed or understand that change is important but believe it is unattainable for various reasons. In this context, mobilizing the motivational systems is crucial to trigger the following stages of change. Several strategies can be employed to achieve this, such as linking health behaviors to values and utilizing nudges.

#### Linking health behaviors to personal values

As previously described, limbic structures regulated by the amygdala assign positive or negative values to experiences. Throughout life, individuals accumulate numerous positive and negative labels, which together form their values. The amygdala activates positive values through its direct connections with the MCLS, which plays an important role in motivation. 33 Therefore, linking new behaviors to an individual's core values and beliefs is critical to move the individual from the non-engagement to the motivational engagement stage.

#### Increasing adaptive behaviors

After initiating a health-behavior change, reinforcing appetitive emotional drives may help to maintain and facilitate the goals. Mechanisms of the MCLS can be activated to enhance adaptive behaviors. 62 Effective strategies include setting clear and achievable goals, training in goal-related behaviors, linking goal achievement to rewards, making the target behavior more enjoyable, structuring the environment to facilitate the desired behavior, activating social support mechanisms, and increasing awareness of the benefits of the new behavior. These strategies align with evidence showing that social and reward-related neurobiological mechanisms tend to reinforce adaptive behaviors. This may involve educational campaigns, personalized feedback, or highlighting positive outcomes in relatable scenarios, ensuring that individuals understand the value and impact of their efforts. 63

#### Reducing maladaptive behaviors

Aversive conditioning, powered by the amygdala, can reduce maladaptive behaviors, making them more challenging or uncomfortable to engage in. 64 Examples of strategies include making high-carb food less accessible or unavailable at home, turning off the internet for some periods, or taking disulfiram for alcohol dependence, using its aversive physiological effects to reduce undesired behavior.

### Managing external factors

Despite its connections to the amygdala, the hypothalamus is significantly influenced by systemic environmental changes, such as caloric, water, thermal, and sleep restrictions. Depending on the situation, the hypothalamus activates stress responses, which may hinder the behavioral change process. Therefore, it is fundamental to adjust the environment to prevent imbalances between the PNS and the SNS, which could predispose relapses in unadaptive behaviors. 34

#### Using nudges

Nudges are interventions that drive people in a particular direction by changing aspects of the choice architecture. They are stimuli, cues, or triggers placed in an environment to increase the motivational salience of the health behaviors. 61 Placing fresh fruits at eye level in grocery stores, for instance, encourages healthier food choices, and displaying step-by-step handwashing guides in public restrooms promotes hygiene. Additionally, apps with automated reminders for physical activity or medication adherence are illustrative examples of how nudges can support behavioral change. 65

### Optimizing voluntary processes

Behavioral change can be viewed as a series of goal-directed behaviors that require significant voluntary control from the PFC. The processes must be optimized to occur effectively.

#### Refining GDBs

Goal-directed behaviors are executed in six stages. The PFC performs these tasks by integrating its functions with those of other brain regions. 34 Behavioral-change approaches can optimize these processes by designing interventions for each stage. Goal-directed behaviors are active processes that must count on the active participation of the individual seeking change ( Figure 5 and Table 5 ).

Stage 1–Goal setting: Establishing achievable and consistent goals is essential to maintain the behavioral-change process. It enhances the motivational drive from the MCLS and sustains the desired behavior.
Stage 2–Planning and sequencing: Multilevel planning, which includes goal progression and sequencing, reduces discomfort caused by uncertainty and guides the change process.
Stage 3–Initiation: The individual evaluates the balance between the necessity and ability to implement the behavioral change, examining its benefits and costs. The behavior will be initiated if the individual perceives the change as worthwhile, feasible, and achievable.
Stage 4–Monitoring and error detection: Implementing strategies for performance monitoring and providing guidance on managing lapses enhances the ability to maintain the new behavior.
Stage 5–Set shifting: Expanding the behavioral repertoire in response to challenging situations and training contingency-based rules enables individuals to maintain target behaviors even under difficult circumstances.
Stage 6–Response inhibition: Self-control in tempting situations can be challenging, but it can also be strengthened by setting goals that align with the individual's values.

**Table 5:** A summary of human behavior physiology and its connection to the Behavior Change Resource Model

Structure	Conditioning type	Connections	Functions	Roles in engagement	Resources
Hypothalamus	Unconditioned	Afferent: locus coeruleus, raphe nuclei, NTS, stria terminalis, amygdala, and PFC; efferent: brainstem (SNS and PNS), amygdala, hippocampus, insula, and PFC	• Vegetative systems; • Autonomic responses; • Maintaining homeostasis	Motivational and executive engagement: a. allostasis bias behavior toward homeostasis recovery; b. reduces voluntary behavioral-control mechanisms	External resources: • fostering conditions to keep homeostatic conditions
Amygdala	Unconditioned aversions and rewards; conditioned avoidance and approaching – respondent conditioning	Afferent: thalamus, midbrain, temporal lobe, PFC; efferent: hypothalamus, hippocampus, nucleus accumbens, and PFC	• Emotional responses; • Value assignment – aversive and appetitive conditioning	Non-engagement: emotional arausal Motivational-engagement: a. Associating health behaviors with positive values; b. Associating unhealthy behaviors with negative values (aversive)	Internal affective resources
MCLS	Unconditioned rewards; xonditioned reward anticipation – operant conditioning	VTA Afferent: amygdala; efferent: NAc, dlPFC, vmPFC, cingulate gyrus, hippocampus, and amygdala; NAc Afferent: cingulate gyrus, anterior insula, PFC, thalamus, hippocampus, amygdala, and VTA; efferent: GPi, amygdala, and HT	• Reward; • Motivation; • Appetitive behaviors toward goals; • Procedural learning	Motivational engagement: a. desire to change; Executive engagement: a. motivation to initiate and sustain change; b. retrieving rewards	Internal affective resources
PFC	Voluntary: mainly new behaviors and situations Conditioned: automating of behavior and cognitive processes	Multiple widespread connections, varying according to subareas (out of scope of the present review)	• Goal-directed behaviors; • Complex reasoning; • Behavioral control; • Theory of mind	Motivational engagement: a. decisional balance; b. goal setting; c. planning; executive engagement: a. initiation; b. monitoring; c. inhibiting impulses	Internal reflective resources

Abbreviations: dlPFC, dorsolateral prefrontal cortex; GPi, globus pallidus internus; HT, hypothalamus; MCLS, mesocorticolimbic system; NAc, nucleus accumbens; NTS, nucleus tractus solitarius; PFC, prefrontal cortex; PNS, parasympathetic nervous system; SNS, sympathetic nervous system; vmPFC, ventromedial prefrontal cortex; VTA, ventral tegmental area.

#### Enhancing emotional modulation

Through its reciprocal connections with the amygdala, the PFC typically reduces aversive emotional impulses that hinder the introduction of new behaviors. Techniques such as self-revaluation and meditation can contribute to this process. 66

## CONCLUSION

In conclusion, in the present review, we describe the neuroscience of behavioral change and maintenance, from neuroanatomy to neurophysiology, and present a detailed discussion of the Behavior Change Resource Model, a motivational theory proposed by Michaelsen and Esch. 11 Furthermore, we interpret this model through a neurobiological lens, mapping each resource category onto relevant brain systems. The integration of these two perspectives, as herein presented, enables a deeper understanding of the mechanisms underlying behavioral change and provides an operational framework to translate theory into clinical practice.
